# IrisDenseNet: Robust Iris Segmentation Using Densely Connected Fully Convolutional Networks in the Images by Visible Light and Near-Infrared Light Camera Sensors

**DOI:** 10.3390/s18051501

**Published:** 2018-05-10

**Authors:** Muhammad Arsalan, Rizwan Ali Naqvi, Dong Seop Kim, Phong Ha Nguyen, Muhammad Owais, Kang Ryoung Park

**Affiliations:** Division of Electronics and Electrical Engineering, Dongguk University, 30 Pildong-ro 1-gil, Jung-gu, Seoul 100-715, Korea; arsal@dongguk.edu (M.A.); rizwanali@dongguk.edu (R.A.N.); k_ds1028@naver.com (D.S.K.); stormwindvn@dongguk.edu (P.H.N.); malikowais266@gmail.com (M.O.)

**Keywords:** iris recognition, iris segmentation, semantic segmentation, convolutional neural network (CNN), visible light and near-infrared light camera sensors

## Abstract

The recent advancements in computer vision have opened new horizons for deploying biometric recognition algorithms in mobile and handheld devices. Similarly, iris recognition is now much needed in unconstraint scenarios with accuracy. These environments make the acquired iris image exhibit occlusion, low resolution, blur, unusual glint, ghost effect, and off-angles. The prevailing segmentation algorithms cannot cope with these constraints. In addition, owing to the unavailability of near-infrared (NIR) light, iris recognition in visible light environment makes the iris segmentation challenging with the noise of visible light. Deep learning with convolutional neural networks (CNN) has brought a considerable breakthrough in various applications. To address the iris segmentation issues in challenging situations by visible light and near-infrared light camera sensors, this paper proposes a densely connected fully convolutional network (IrisDenseNet), which can determine the true iris boundary even with inferior-quality images by using better information gradient flow between the dense blocks. In the experiments conducted, five datasets of visible light and NIR environments were used. For visible light environment, noisy iris challenge evaluation part-II (NICE-II selected from UBIRIS.v2 database) and mobile iris challenge evaluation (MICHE-I) datasets were used. For NIR environment, the institute of automation, Chinese academy of sciences (CASIA) v4.0 interval, CASIA v4.0 distance, and IIT Delhi v1.0 iris datasets were used. Experimental results showed the optimal segmentation of the proposed IrisDenseNet and its excellent performance over existing algorithms for all five datasets.

## 1. Introduction

In the last two decades, biometrics have been completely incorporated into our daily life. Owing to research efforts, biometrics are now adopted to various applications such as person authentication and identification at airports or in national databases. Biometrics in both physiological and behavioral forms are delivering an efficient platform for security metrics [1]. Physiological biometrics include fingerprint recognition [2], finger vein pattern recognition [3], face recognition [4], iris recognition [5], and palmprint recognition [6]. Iris recognition has been proven as innovative reliable biometric widely used in security, authentication, and identification systems [7].

Iris recognition shares the following advantages of secure biometrics: iris features are unique even in the case of twins, left eye iris features of an individual are different from right eye iris features, iris features are naturally complex to be created artificially, and iris features are permanent and remain same throughout a human’s lifespan [8].

Innovative research in iris recognition is related to iris applications in mobile and handheld devices. To provide reasonable security to mobile devices, iris patterns are being used to replace passwords or lock-patterns, which makes the device more user-friendly and convenient. Currently, mobile and handheld devices are mainly secured with fingerprint security, but there have been spoofing cases and it is inconvenient to develop separate fingerprint scan hardware in the smart device and rather easy to implement an iris-based system using a frontal camera [9]. For individuals working in intensive security departments, usage of personal digital assistant (PDA) and notepads is much needed, but this essential hardware lacks a security mechanism, which might lead to fraudulent usage. Therefore, building light algorithms that can be efficiently used with mobile devices is a new-era requirement [10]. The major challenge here is to achieve high performance on a mobile platform because of limitation of space, power, and cost of the system. In conclusion, each stage of iris recognition should be designed such that it reduces process time with improved reliability of recognition with less user cooperation in different light environments [11].

Most iris recognition systems consist of five elementary steps: iris image acquisition, pre-processing, iris boundary segmentation, iris feature extraction, and matching for authentication or identification. The acquired image is pre-processed to eliminate the noise captured in the first step. Then, in the third step, iris boundaries are segmented using various algorithms to extract the iris from the image. In the fourth step, the iris features are extracted from the segmented image, and usually, a code is generated with the template using the encoding scheme. Finally, in the last step, the codes are matched for user authentication or identification [12].

### Why Is Iris Segmentation Important?

There are two types of image acquisition environments: ideal and non-ideal. In ideal environments, the iris area is not affected by eyelids and eyelashes, and images are under ideal light conditions. Therefore, recognition rates are high and conventional methods can perform well. On the other hand, in non-ideal environments, the images contain blurs, off-angles, non-uniform light intensities, and obstructions. Therefore, in both ideal and non-ideal environments, a real iris boundary without occlusion is required for better error-free features, so a segmentation algorithm is needed to separate each type of noise from the iris image and provide a real iris boundary even in non-ideal situations [13]. A good segmentation algorithm significantly affects the accuracy of the overall iris recognition system and can handle errors generated by occlusions of eyelashes, motion blurs, off-angle irises, specular reflections, standoff distances, eyeglasses, and poor illuminations [14]. Previous research showed that the error generated in the iris segmentation stage is propagated in all subsequent stages of recognition [15]. Proenca et al. analyzed 5000 images of UBRIS, CASIA, and ICE databases, and concluded that the incorrect segmentation in horizontal and vertical directions affects the recognition errors [16].

## 2. Related Work

The present schemes for iris segmentation can be categorized into five main implementation approaches: iris circular boundary detection without eyelid and eyelash detection, iris circular boundary detection with eyelid and eyelash detection, active contour-based iris segmentation, region growing and watershed-based iris segmentation, and finally, the most elegant, deep-learning-based iris segmentation.

### 2.1. Iris Circular Boundary Detection without Eyelid and Eyelash Detection

These methods are usually developed for ideal environments and consider the iris and pupil as a circle and do not deal with occlusions. Hough transform (HT) detects a circular iris boundary in iris images, and determines the circularity of the objects based on edge-map voting considering the given limits of the iris or pupil radii, well known as Wilde’s method [17]. Many variants of Daugman’s method using iris circular boundary detection have been developed [18]. Khan et al. proposed a gradient-based method in which 1-D profile lines were drawn on the iris boundary, the gradient was computed on each profile line, and the maximum change represented the iris boundary [19]. Ibrahim et al. used a two-stage method for a pupillary boundary circular moving window accompanied by probability, where the iris boundary was detected using the gradient on rows with the pupil [20]. Huang et al. used radial suppression-based edge detection and thresholding to detect the iris circular boundary [21]. Jan et al. used pre-processing for specular reflection and detected the boundaries using HT assisted by gray-level statistics, thresholding, and geometrical transforms [22]. Ibrahim et al. proposed an automatic pupil and iris localization method in which the pupillary boundary was detected by automatic adaptive thresholding and the iris boundary was detected by the first derivative of each row with the pupil [23]. Umer et al. first found the iris inner boundary based on restricted HT. For finding the outer iris boundary, inversion transform, image smoothing, and binarization were performed, and finally, restricted HT was computed for finding the external boundary [24].

### 2.2. Iris Circular Boundary Detection with Eyelid and Eyelash Detection

In this sub-section, we explain the conventional methods which initially compute the iris as a circular object but try to approximate the real iris boundary by using other methods such as eyelash and eyelid detection. Daugman adopted the integro-differential operator to approximate the iris circular boundaries, and detected the eyelid with eyelash by using an additional algorithm [25]. Jeong et al. proposed an effective approach using two circular edge detector in combination with AdaBoost for inner and outer iris boundary detection. To reduce the error, eyelid and eyelash detection was performed [26]. Parikh et al. first found the iris boundary based on color-clustering. To deal with off-angle iris images, he detected two circular boundaries from both right and left of the iris, where the overlapped area of these two boundaries represented the outer iris boundary [27]. Pundlik et al. used the graph cut-based approach for iris segmentation in non-ideal cases, where the eyelashes were separated from the images by image texture using a Markov random field. A energy minimization scheme based on a graph cut was used to segment the iris, pupil, and eyelashes [28]. Zuo et al. proposed a method of non-ideal iris segmentation where non-ideal factors such as off-angle, blur, contrast, and unbalanced illumination were detected and compensated for each eye separately. Both pupillary and iris boundaries were detected by a rotated ellipse fitting in combination with occlusion detection [29]. Hu et al. proposed a novel method for color iris segmentation based on the fusion strategy using three models and by choosing the best strategy automatically, where limbic boundaries were segmented using Daugman’s integrodifferential operator with high-score fitting based on the iris center [30].

### 2.3. Active Contours for Iris Segmentation

Active contours are a step toward detecting the real boundary. Shah et al. used the geodesic active contour-based approach to extract the iris contour from the surrounding structures. Because the active contour can assume shapes and segment multiple objects, iteratively fashioned boundaries of the iris are found by the guidance of global and local properties of the image [31]. Koh et al. used the combination of active contour and HT to locate the outer and inner iris boundaries for non-ideal situations [32]. Abdullah et al. proposed an accurate and fast method for segmenting the iris boundary by using Chan–Vese active contour and morphology [33]. They proposed an active contour-based fusion technique with shrinking and expanding iterations. A pressure force applied to the active contour model was used to make the method robust. The non-circular iris normalization technique was adopted as a new closed eye detection method [34].

### 2.4. Region Growing/Watershed-Based Iris Segmentation Methods

These types of method are similar to those used for detecting the true iris boundary. Tan et al. proposed a region growing-based approach. After the rough iris and non-iris boundaries are found, a novel integro-differential constellation is constructed with a clustered region growing algorithm. For accurate detection of inner and outer iris boundaries, eight-neighbor connection and point-to-region distance were evaluated [35]. Patel et al. proposed a region growing of pupil circle and the method based on binary integrated curve of intensity to reduce the difficulties created by non-ideal segmentation conditions. The approach avoided the eyelid portion, and hence, was close to the real boundary [36]. Abate et al. proposed an iris segmentation method for the images captured in visible light on mobile devices. To detect the iris boundary in a noisy environment, he used a watershed transform named watershed-based iris detection (BIRD). The watershed algorithm is a growing process performed generally on the gradient image. To reduce the number of watershed regions, the seed selection process is used [37].

### 2.5. CNN for Iris Segmentation

To solve the problems of previous methods and lessen the computational burden of pre- and post-processing, convolutional neural network (CNN)-based iris segmentation is proposed. CNN provides a strong platform for segmentation tasks such as brain tumor segmented using several kernels [38]. So far, iris segmentation has been rarely researched using CNN whereas it is mostly used for iris recognition purposes. Ahuja et al. proposed two convolution-based model to verify a pair of periocular images including the iris patterns [39]. Zhao et al. proposed a new semantic-assisted convolutional neural network (SCNNs) to match the periocular images [40]. Al-waisy et al. proposed an efficient real-time multimodal biometric system for iris detection [41]. Gangwar et al. proposed DeepIrisNet for cross-sensor iris recognition [42]. Lee et al. proposed a method for iris and periocular recognition based on three CNNs [43].

Considering the iris segmentation, Liu et al. proposed two modalities using fully convolutional networks (FCNs), where multi-scale FCNs (MFCNs) and hierarchical CNNs (HCNNs) were used to find the iris boundaries in non-cooperative environments without using handcrafted features [44]. Arsalan et al. used a two-stage deep-learning-based method to identify the true iris boundary, and modified HT to detect a rough iris boundary, which is provided to the second stage, which utilizes the deep learning model to identify the 21 × 21 mask as an iris or non-iris pixel [45]. As CNN-based segmentation schemes require considerable labeled data, Jalilian et al. proposed a new domain adaption method for CNN training with a few training data [46]. These schemes have better accuracies compared to the previous methods, but the accuracy of iris segmentation can be further enhanced.

To address the issues of accurate segmentation without prior pre-processing and to develop a robust scheme for all types of environments, this study presents a densely connected fully convolutional network (IrisDenseNet)-based approach to detect an accurate iris boundary with better information gradient flow due to dense connectivity.

Table 1 shows a comparison between prevailing methods and the proposed IrisDenseNet.

## 3. Contribution

This study focuses on an iris image of low quality in non-cooperative scenarios where segmentation is quite difficult with the existing methods. The proposed IrisDenseNet accurately identifies the iris boundary even in low qualified iris images, such as side views, glasses, off-angle eye images, rotated eyes, non-uniform specular reflection, and partially opened eyes. Following are the five novelties of this study:

- IrisDenseNet is an end-to-end segmentation network that uses the complete image without prior pre-processing or other conventional image processing techniques with the best information gradient flow, which prevents the network from overfitting and vanishing gradient problem.
- This study clarifies the power of dense connectivity with a visual difference between the output feature maps from the convolutional layers for dense connectivity and normal connectivity.
- IrisDenseNet is tested with noisy iris challenge evaluation part-II (NICE-II) and various other datasets, which include both visible light and NIR light environments of both color and greyscale images.
- IrisDenseNet is more robust for accurately segmenting the high-frequency areas such as the eyelashes and ghost region present in the iris area.
- To achieve fair comparisons with other studies, our trained IrisDenseNet models with the algorithms are made publicly available through [47].

## 4. Proposed Method

### 4.1. Overview of the Proposed Architecture

**Figure 1:**
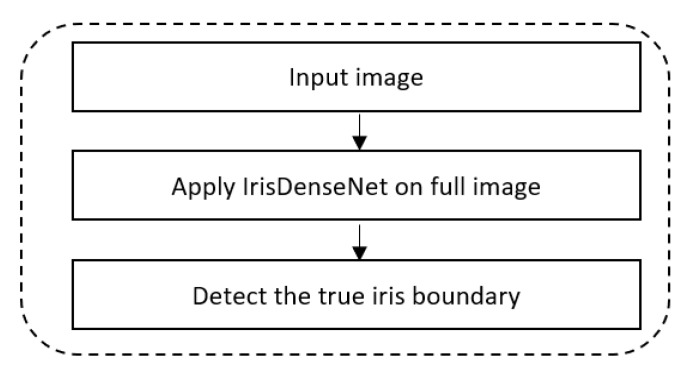
Flowchart of the proposed method.

**Figure 2:**
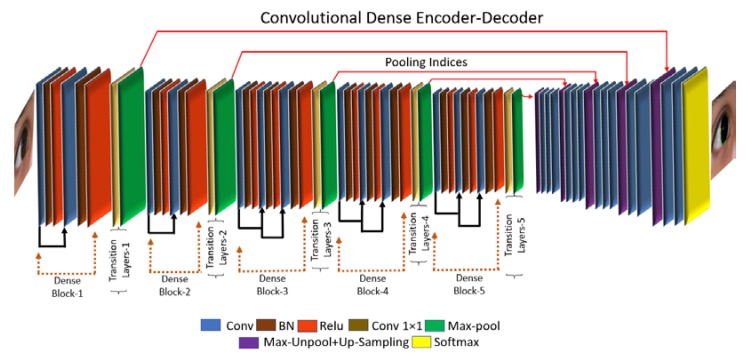
Overview of the proposed method.

Figure 1 shows the overall flowchart of the proposed IrisDenseNet for iris end-to-end segmentation. The input image is given to the IrisDenseNet fully convolutional network without any pre-processing. The network applies the convolutions and up-sampling via pooling indices, and on the basis of learning, provides semantic segmentation mask for a true iris boundary. Figure 2 shows an overview of the proposed IrisDenseNet model for iris segmentation with dense connectivity. The network has two main parts: densely connected encoder and SegNet decoder. In Figure 2, convolutional layers (Conv), batch normalization (BN) and rectified linear unit (ReLU) indicate a convolution layer, a batch normalization layer, and a rectified liner unit layer, respectively. To ensure the information flow between the network layers, dense connectivity is introduced by the direct connections from any layer to all subsequent layers in a dense block. In this study, overall, five dense blocks are used, which are separated by transition layers (a combination of Conv 1 × 1 and max-pool layers).

### 4.2. Iris Segmentation Using IrisDenseNet

In the last decade, CNN has been proved as the most powerful tool for image-related tasks using deep learning. CNN delivers good performances in computer vision applications such as human detection [48], open and close eye detection [49], gender recognition [50], pedestrian detection [51], banknote classification [52], appearance-based gaze estimation [53], and object detection using faster R-CNN [54]; more CNN applications can be found in [55].

To ensure the reliability and accuracy of CNNs, this paper proposes a combination of two foundation methods: (i) densely connected convolutional networks with strengthening feature propagation (DenseNet) [56] and (ii) SegNet [57] deep convolutional encoder–decoder network. SegNet is a practical fully convolutional network for pixel-wise semantic segmentation, which uses the encoder of a 13-layered VGG16 identical core network with fully connected layers removed. The decoder up-samples the low-resolution input feature maps with pooling indices. In our study, we use the SegNet-Basic decoder. SegNet uses a VGG16 identical network owing to the drawback of overfitting and vanishing gradient. Densely connected convolutional network (DenseNet) [56] is proved to be more robust than VGG-net [58] with better information gradient flow due to dense connectivity. In DenseNet, each convolutional layer is connected to all convolutional layers in a feed-forward fashion, which is very useful for strengthening the feature propagation in the subsequent layers in a dense block, as shown in Figure 2.

**Figure 3:**
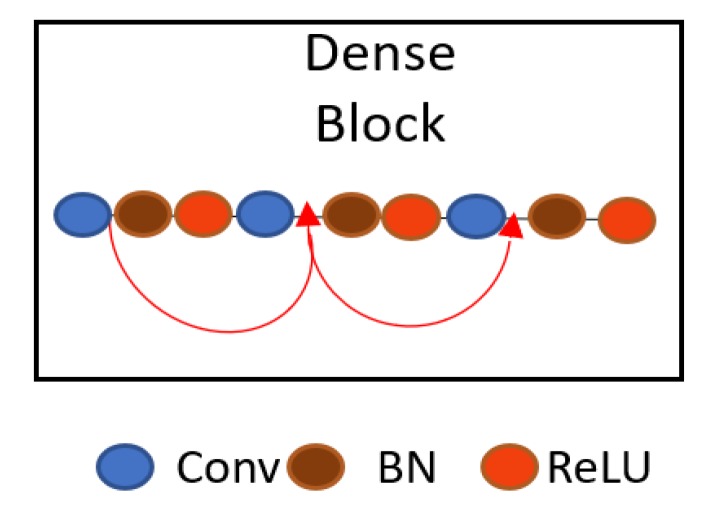
Dense connectivity within the dense block by feature concatenation.

**Figure 4:**
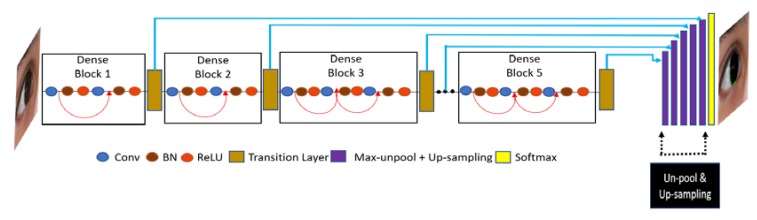
Overall connectivity diagram of IrisDenseNet dense encoder–decoder.

Through this connectivity, IrisDenseNet exploits the network potential through the feature reused from the previous layer, which results in enhanced efficiency. Direct connection is basically the feature concatenation achieved by concatenation layers with multiple inputs and one output. Figure 3 shows one dense block separately, and includes the basic convolutional layers (Conv), batch normalization (BN) and rectified linear unit (ReLU), which includes the connections of the layers via concatenation. The pooling indices that are provided after each dense block by max-pooling in the transition layer in the decoder part are shown in Figure 4. These pooling indices are used to un-pool and up-sample for pixel-wise semantic segmentation of iris and non-iris boundaries. The IrisDenseNet encoder and decoder are explained in detail in Section 4.2.1 and Section 4.2.2, respectively.

#### 4.2.1. IrisDenseNet Dense Encoder

Owing to the advantages of dense connectivity explained in Section 4.2, this study is based on a densely connected encoder in which five dense blocks are used to improve the performance. Finally, with the SegNet decoder (described in Section 4.2.2), a densely connected convolutional network for iris segmentation (IrisDenseNet) is created. The IrisDenseNet dense encoder consists of 18 convolutional layers, including five 1 × 1 Conv layers used as the bottleneck layer in transition layers after each dense block, which is useful in reducing the number of input feature maps for computational efficiency. Figure 4 shows the complete dense connectivity with the operation of transition layers. The transition layers are basically a combination of Conv 1 × 1 and max-pooling, separate two adjacent dense blocks. The IrisDenseNet have the following five key differences compared to DenseNet [56].

- DenseNet is using 3 dense blocks for CIFAR and SVHN datasets and 4 dense blocks for ImageNet classification whereas IrisDenseNet uses 5 dense blocks for each dataset.
- In DenseNet, all dense blocks have four convolutional layers [56], whereas IrisDenseNet has two convolutional layers in the first two dense blocks and 3 convolutional layers for the remaining dense blocks.
- In IrisDenseNet, the pooling indices after each dense block are directly fed to the respective decoder block for the reverse operation of sampling.
- In DenseNet, fully connected layers are used for classification purpose, but in order to make the IrisDenseNet fully convolutional, the fully connected layers are not used.
- In DenseNet, the global average pooling is used in the end of the network whereas in IrisDenseNet, global average pooling is not used to maintain the feature map for decoder operation.

The dense connectivity has following advantages over simple connections:

- It substantially reduces the vanishing gradient problem in CNNs, which increases the network stability.
- Dense connectivity in the encoder strengthens the features flowing through the network.
- It encourages the feature to be reused due to direct connectivity, so the learned features are much stronger than those of normal connectivity.

A detailed layer-wise structure is provided in Table 2 for better understanding, which shows that in a dense block, due to feature map concatenation, the output feature size for all layers in a corresponding dense block always remains the same, which guarantees strong features. As shown in Figure 4, the features through the red and half-circle lines (including arrows) in each dense block are concatenated with the output features of convolution layer, and this concatenation layer is implemented by depthConcatenationLayer() function. In details, there are two concatenation layers in each dense blocks 3, 4, and 5. For example, the first concatenation layer (Cat-3 of Table 2) obtains the output features by concatenating the output features of the 1st convolution layer (Conv-3_1 of Table 2) with the output features of the 2nd convolution layer (Conv-3_2 of Table 2). The second concatenation layer (Cat-4 of Table 2) obtains the output features by concatenating the output features of the first concatenation layer (Cat-3 of Table 2) with the output features of the 3rd convolution layer (Conv-3_3 of Table 2). Same procedure is applied to dense blocks 4 and 5, also.

#### 4.2.2. IrisDenseNet Decoder

As described in Section 4.2.1, the SegNet-Basic decoder is used to up-sample the dense feature provided by the dense encoder in this study. The decoder basically utilizes the pooling indices along with dense features, and the feature maps are again passed through convolution filters for a reverse process to the encoder to obtain the segmentation mask with same size as that of the input. In the end, each pixel is classified by the soft-max function independently as iris or non-iris via the pixel classification layer.

Figure 4 shows the overall dense encoder–decoder operation. Note that the decoder un-pools and up-samples the pooling in reverse order. The dense features from the last dense block (Dense block 5) relate to the first decoder layers, whereas those from the first dense block relate to the last decoder layers. Table 2 shows that the output feature size is smallest with dense block 5 and largest with dense block 1. Note that the decoder gets the pooling indices from the transition layers, which are a combination of the 1 × 1 convolution layer and a max-pooling layer. The decoder is explained in detail in [57].

## 5. Experimental Results

### 5.1. Experimental Data and Environment

**Figure 5:**
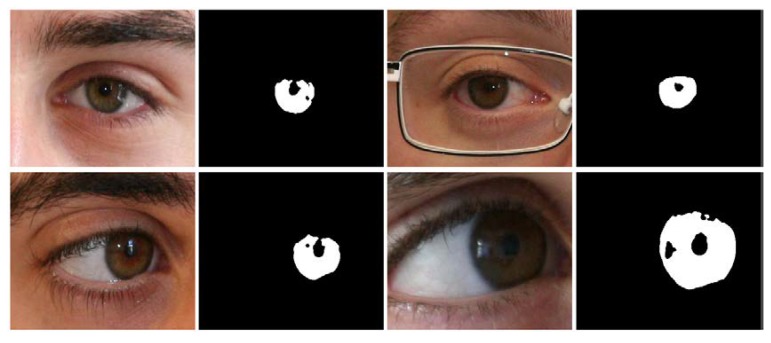
Noisy iris challenge evaluation part-II (NICE-II) sample images with corresponding ground truths.

In this study, the NICE-II dataset is used as iris images in visible light environment. The NICE-II database is used for NICE-II competition for iris recognition [59], and consists of 1000 enormously noisy iris images selected from the UBIRIS.v2 database. The database contains 400 × 300 pixel images captured from a Canon EOS 5D camera of 171 classes walking 4–8 m away from the camera. The database includes intruded difficulties such as motion blurs, eyelash and eyelid occlusions, glasses, off-angles, non-uniform light illuminations, and partially captured iris images with irregular rotations. The ground-truth images are publicly available with the database for comparison. In Figure 5, sample images from the NICE-II database are shown with corresponding ground-truth images.

In this study, half of the total NICE-II images are used for training and the remaining are used for testing purposes based on two-fold cross-validation. To ensure the accuracy of the segmentation, data augmentation is performed as described in Section 5.2. The training and testing of IrisDenseNet are performed on Intel^®^ Core™ i7-3770K CPU @ 3.50 GHz (4 cores) with 28 GB RAM and NVIDIA GeForce GTX 1070 (1,920 Cuda cores) with graphics memory of 8 GB (NVIDIA, Santa Clara, CA, USA) [60] using MATLAB R2017b [61]. We did not use any pretrained models of ResNet-50, Inception-v3, GoogleNet, and DenseNet in our research. Instead, we implemented our IrisDenseNet by using MATLAB functions. In addition, we performed the training our whole network of IrisDenseNet (training from the scratch) with our experimental dataset.

### 5.2. Data Augmentation

In this study, semantic segmentation of the iris boundary using the full image is proposed, which is much dependent on considerably large amount of image and ground-truth data. Owing to the limited number of images, data augmentation is used to increase the volume of data for training. In this study, each image of training data is augmented 12 times. The augmentation is performed in the following ways:

- Cropping and resizing with interpolation
- Flipping the images only in horizontal direction
- Horizontal translation
- Vertical translation

### 5.3. IrisDenseNet Training

To segment the iris in a challenging situation, no pre-processing is performed for training. If models that are very deep with the linearity of ReLU are used, the weight initialization with a pre-trained model helps in convergence when training from scratch [62]. Using the same concept, weights are initialized by VGG-16 trained on ImageNet [63] for better training. To train all datasets, a fixed learning rate of 0.001 is used with stochastic gradient descent, which helps to reduce the difference between the desired and calculated outputs by a gradient derivative [64] with a weight decay of 0.0005.

IrisDenseNet is trained using 60 epochs. The mini-batch size is kept 4 with shuffling for the NICE-II dataset. The cross-entropy loss proposed in [65] is used as an unbiased function to train the network, where the loss is calculated over all pixels that are available in a mini-batch acceding to classes (iris and non-iris).

**Figure 6:**
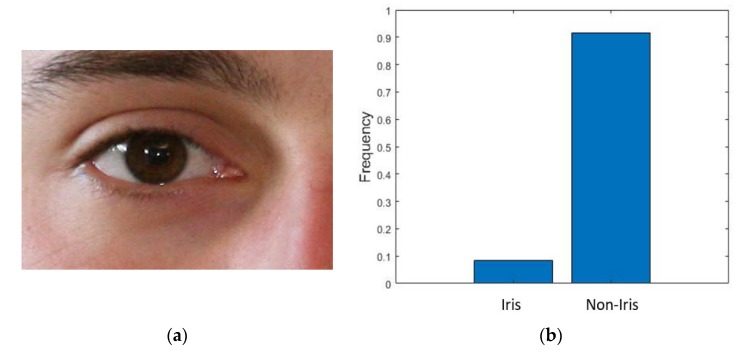
Difference in frequency between iris and non-iris classes. (**a**) NICE-II original input image. (**b**) Difference in frequency of iris and non-iris pixels in NICE-II training dataset.

Considering the iris image from the NICE-II database in Figure 6a, the iris size is usually much smaller than the non-iris areas, from which we can deduce that the number of non-iris pixels is much larger than that of the iris pixels. Therefore, during training over a dataset, there can be a large difference of frequency in each class, as shown in Figure 6b.

The frequencies of the iris and non-iris pixels indicate that the non-iris class dominates much during training, so there is a need to maintain a balance between these two classes during training. This type of frequency balancing is used when some classes are underrepresented in the training data. In this study, median frequency balancing [66] is used, where a weight is assigned to the cross-entropy loss. This weight is calculated from the training data by median class frequency (for both iris and non-iris classes) by the following given formulas:

(1) $$W_{c1}=\frac{Median\_freq}{f_{c1}}\;\text{and}\;W_{c2}=\frac{Median\_freq}{f_{c2}}\;$$

In Equation (1), $W_{c1\;}$ and $W_{c2\;}$ are the class weights for iris and non-iris classes, respectively, $f_{c1}$ indicates the number of iris pixels over the total number of pixels in images, and $f_{c2}$ indicates the number of non-iris pixels over the total number of pixels in images. *Median_freq* is the median of $f_{c1}$ and $f_{c2}$. With frequency balancing, a weight smaller than 1 is assigned to a larger (non-iris) class and a weight larger than 1 is assigned to a smaller (iris) class in the cross-entropy loss during training.

**Figure 7:**
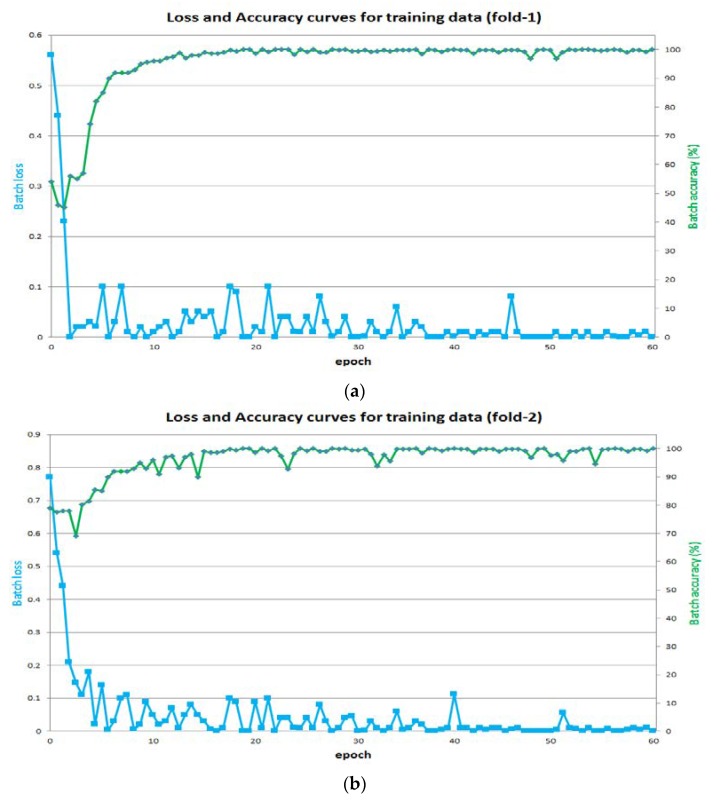
Training accuracy and loss curves from (**a**) 1st-fold cross-validation and (**b**) 2nd-fold cross-validation.

Figure 7a,b show the accuracy and loss curves for training from the 1st- and 2nd-fold cross-validation, respectively. The x-axis for each curve shows the number of epochs and the y-axis shows the training accuracy and loss. During the training, it is important to achieve accuracy close to maximum and a loss close to the minimum. The loss is dependent on the learning rate, so the learning rate is empirically found by conducting an experiment to achieve the minimum loss. With an increased learning rate, the training loss can decrease dramatically, but it is not certain that the training would converge to the valley point for the loss. In our proposed method, we achieve a training accuracy approaching 100% and a loss approaching 0%. As described in Section 3, our trained models with algorithms are made publicly available through [47] to make fair comparisons with other studies.

### 5.4. Testing of IrisDenseNet for Iris Segmentation

To obtain the segmentation results from the proposed IrisDenseNet, the input image is provided to the trained model and there is no pre-processing involved during training and testing. The input image is passed through the dense encoder and decoder in a forward fashion. The output of the trained model is a binary segmentation mask, which is used to generate and evaluate the segmentation results by using our trained model. The performance of the proposed IrisDenseNet is evaluated using the NICE-I evaluation protocol [67], which is being used by many researchers to evaluate the segmentation performance. *E_i_* is computed by exclusive-OR (XOR) between the resultant image ($I_{i}\left(m^{\prime },n^{\prime }\right)$) and ground-truth image ($G_{i}\left(m^{\prime },n^{\prime }\right)$) given as

(2) $$E_{i}=\frac{1}{m\times n}\underset{\left(m^{\prime },n^{\prime }\right)}{\sum }\;I_{i}\left(m^{\prime },n^{\prime }\right)⊗\;G_{i}\left(m^{\prime },n^{\prime }\right)\;$$

where m×n is the image size (by width and height of the image). For each image, $E_{i}$ is calculated as the pixel classification accuracy. The overall average segmentation error $E_{a}$ is calculated by averaging the classification error ($E_{i}$) over all images in the database:

(3) $$E_{a}\;=\frac{1}{t}\;\underset{i}{\sum }E_{i}$$

where t represents the total number of images to be evaluated. The value of $E_{a}$ always lies within [0, 1]. If $E_{a}$ is close to “0,” it shows the minimum error, whereas if $E_{a}$ is close to “1,”it shows the largest error.

#### 5.4.1. Result of Excessive Data Augmentation

Data augmentation is a method of increasing the training data for better accuracy, but this accuracy is strongly dependent upon the augmentation type. The data augmentation varies with the application, so in case of iris, it is experimentally observed that excessive data augmentation results in reduced accuracy, as shown in Table 3. As explained in Section 5.2, each image of training data was augmented 12 times in our study. In the case of excessive data augmentation, each image of training data was augmented 25 times. Moreover, if we augment the data by changing the contrast and brightness of the iris image, the overall performance degrades in terms of segmentation accuracy as shown in Table 3. The reason why the lower accuracy is obtained by excessive data augmentation is due to the overfitting of training. The reason why the lower accuracy is obtained by data augmentation by changing the contrast and brightness is that the augmented data based on this scheme do not reflect the characteristics of testing data.

#### 5.4.2. Iris Segmentation Results Obtained by the Proposed Method

**Figure 8:**
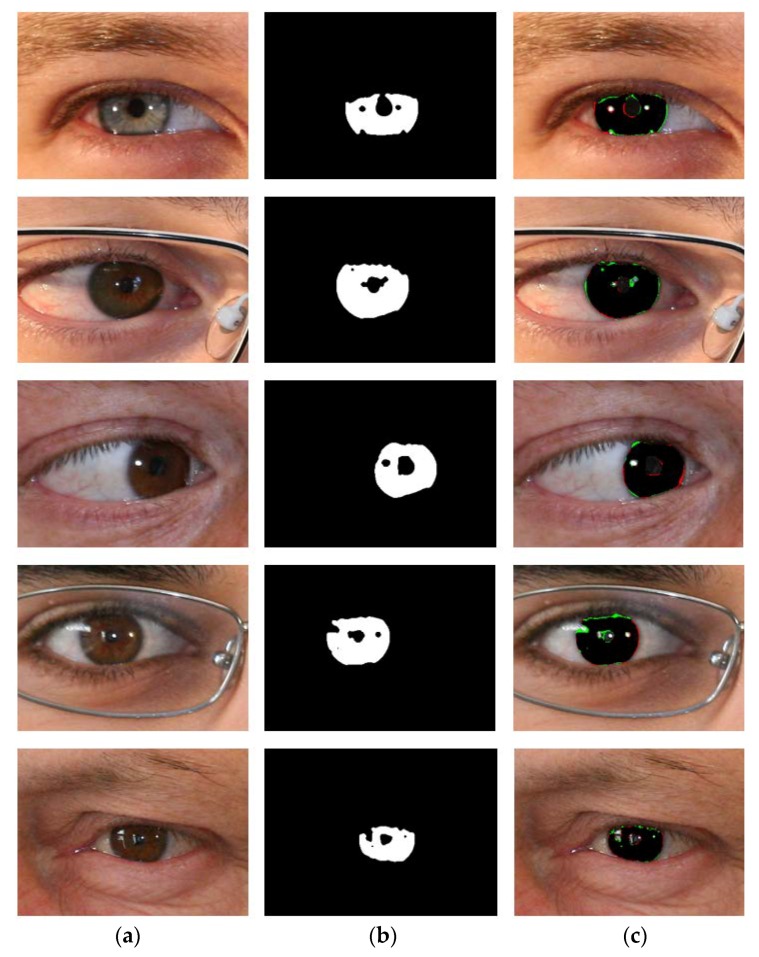
Examples of NICE-II good segmentation results obtained by IrisDenseNet. (**a**) Original image. (**b**) Ground-truth image. (**c**) Segmentation result obtained by IrisDenseNet (The false positive and negative errors are shown in green and red, respectively. The true positive case is shown in black).

**Figure 9:**
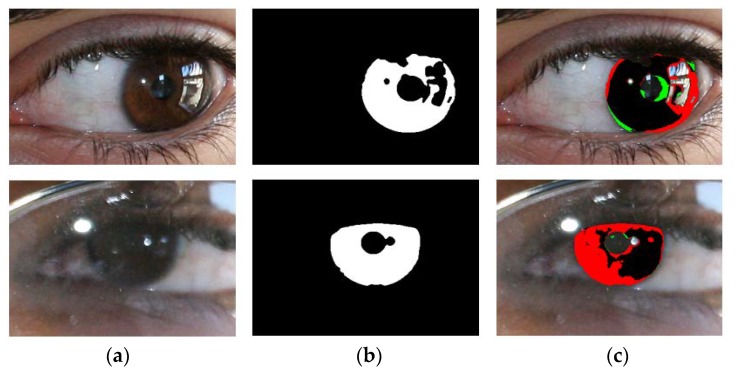
Examples of incorrect iris segmentation by our method. (**a**) Original input images. (**b**) Ground-truth images. (**c**) Segmentation results (The false positive and negative errors are presented as green and red, respectively. The true positive case is shown in black).

Figure 8 shows the good segmentation results obtained by IrisDenseNet for the NICE-II dataset. As explained in Section 5.4, the performance of the proposed method is measured as $E_{a},$ which is the average error for the database. To pictorially represent the result, two error types, false positive and false negative, are defined. The former represents the error of a non-iris pixel being misclassified as an iris pixel, whereas the later represents the error of an iris pixel being misclassified as a non-iris pixel. The false positive and negative errors are represented in green and red, respectively. The true positive case is that the iris pixel is correctly classified as an iris pixel, which is represented in black. As shown in Figure 8 and Figure 9, the false positive error is caused by the eyelash pixel or pixel close to the reflection noise or pupil, whose value is similar to that of the iris pixel, whereas the false negative error is caused by reflection noise caused from glasses or with a dark iris area.

#### 5.4.3. Comparison of the Proposed Method with Previous Methods

In this section, experimental results of the NICE-II dataset are given by IrisDenseNet on the basis of $E_{a}$ of Equation (3), and SegNet-Basic is tested on the same dataset for comparison. To clarify the power of dense features, a visual comparison of convolutional features by SegNet and IrisDenseNet is also provided in Section 6.1. Table 4 represents the comparative analysis of NICE-II with existing methods based on the NICE-I evaluation protocol. Note that SegNet-Basic is a general semantic segmentation method used for road scene segmentation (road, cars, building, pedestrian, etc.) and the dataset of CamVid road scene segmentation [57,68] with 11 classes. For comparison, the number of outputs for the original SegNet-Basic is changed from 11 to 2 iris and non-iris classes. As shown in Table 4, we can confirm that our IrisDenseNet shows an error lower than those generated in the previous method. The reason why SegNet-Basic shows lower accuracy than our IrisDenseNet is that the scheme of re-using features through dense connections is not adopted in SegNet-Basic.

#### 5.4.4. Iris Segmentation Error with Other Open Databases

This study includes additional experiments with four other open databases: Mobile iris challenge evaluation (MICHE-I) [80], CASIA-Iris-Interval (CASIA v4.0 interval) [81], CASIA-Iris-Distance (CASIA v4.0 distance) [81], and IIT Delhi (IITD) Iris Database (v1.0) [82]. Experiments conducted in different environments such as visible light and NIR light show the robustness of the proposed method.

MICHE-I is a challenging dataset captured with mobile devices to ensure the developing algorithms in non-ideal difficult situations. This database is collected using three smartphones: iPhone5 with 8 MP (72 dpi) back camera and 1.2 MP (72 dpi) frontal camera, Samsung Galaxy S4 with 13 MP (72 dpi) back camera and 2 MP (72 dpi) frontal camera, Samsung Galaxy Tab2 with 0.3 MP frontal camera (with pixel resolutions of 1536 × 2048, 2322 × 4128, and 640 × 480 for iPhone5, Galaxy S4, and Galaxy Tab2, respectively). This database is collected with 195 subjects over two visits with a distance 5–25 feet under non-ideal conditions (indoor and outdoor), including motion blur, occlusions, off-angle, and non-uniform illuminations. Ground-truth data are not provided in MICHE-I database; therefore, we use the images whose ground truth can be correctly identified by their provided algorithm [74] according to the instruction of MICHE. The total numbers of images in sub-datasets from iPhone5, Galaxy S4, and GalaxyTab2 are 611, 674, and 267, respectively. In detail, the numbers of images for training (testing) are 311 (300), 340 (334), and 135 (132) in the sub-datasets from iPhone5, Galaxy S4, and GalaxyTab2, respectively.

**Figure 10:**
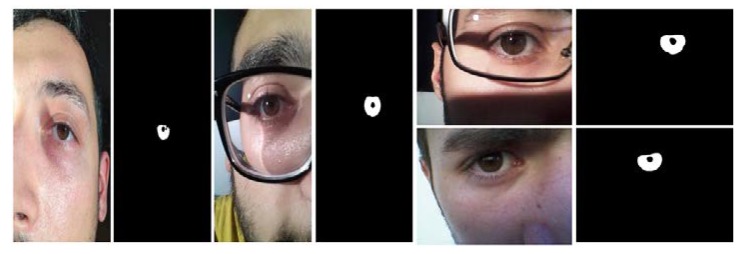
Mobile iris challenge evaluation (MICHE-I) sample images with corresponding ground truths.

Figure 10 shows the sample images for MICHE-I with corresponding ground truths for iPhone5 (left image), Samsung Galaxy S4 (middle image), and Samsung Galaxy Tab2 (right images).

**Figure 11:**
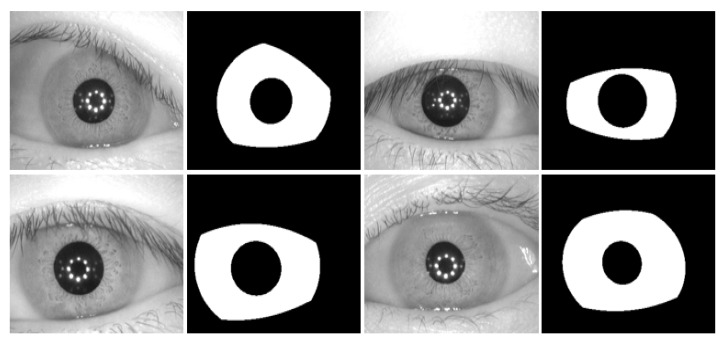
The institute of automation, Chinese academy of sciences (CASIA) v4.0 interval sample images with corresponding ground truths.

The CASIA v4.0 interval dataset is captured with a CASIA self-developed camera with a special circular NIR LED array and appropriate luminous intensity for iris imaging. Owing to this suitable camera setup and novel design, very clear iris images with very detailed features are captured. The images are grayscale and have a pixel resolution of 320 × 280. The ground truth for the CASIA v4.0 interval database is provided by the IRISSEG-EP [83]. Figure 11 shows the sample images and corresponding ground truth.

**Figure 12:**
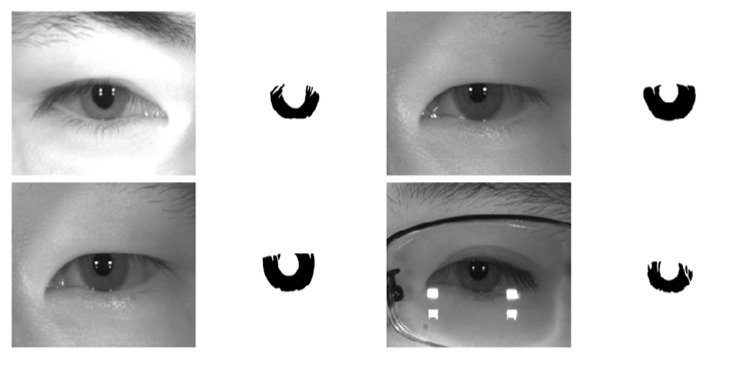
CASIA v4.0 distance sample images with corresponding ground truths.

The CASIA v4.0 distance database contains iris images from a long-range multimodal biometric image acquisition and recognition system (LMBS). These images are captured using a high-resolution NIR camera. This database contains 2567 images from 142 subjects captured from 3 m distance from the camera. The ground truth for CASIA v4.0 distance is not publicly available. Instead, 400 iris images from 40 subjects are manually labeled and publicly available for research purposes through [47]. Figure 12 shows the sample images for CASIA v4.0 distance with provided corresponding ground truths.

**Figure 13:**
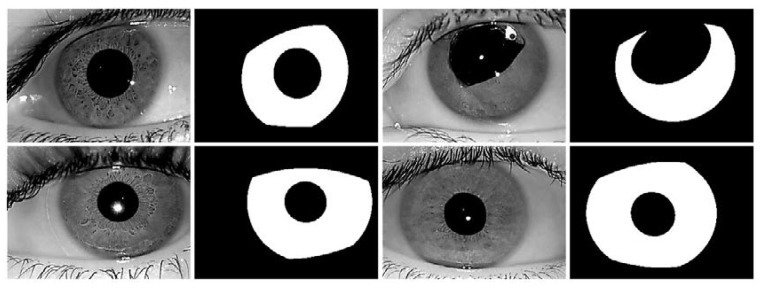
IIT Delhi (IITD) v1.0 sample images with corresponding ground truths.

The IITD Iris database consists of 2240 iris image from 224 subjects with a JPC1000 digital CMOS camera in NIR light environment with a pixel resolution of 320 × 240 [82]. This image database is taken in an indoor controlled environment with user cooperation for a frontal view of iris, so no off-angle iris is involved in this database. The ground truth for IITD is also provided by the IRISSEG-EP [83]. Figure 13 shows the sample images and corresponding ground truth for IITD iris database.

**Figure 14:**
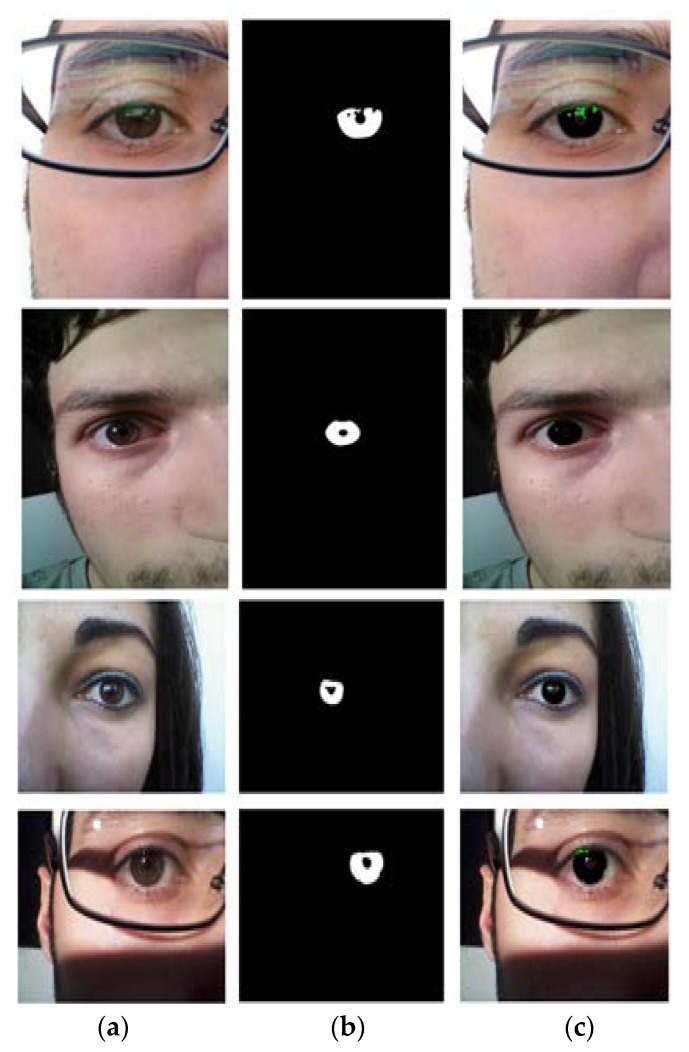
Examples of correct segmentation results in MICHE-I database by our method. (**a**) Original image. (**b**) Ground-truth image. (**c**) Segmentation result by IrisDenseNet (The false and negative errors are presented as green and red, respectively. The true positive case is presented as black).

**Figure 15:**
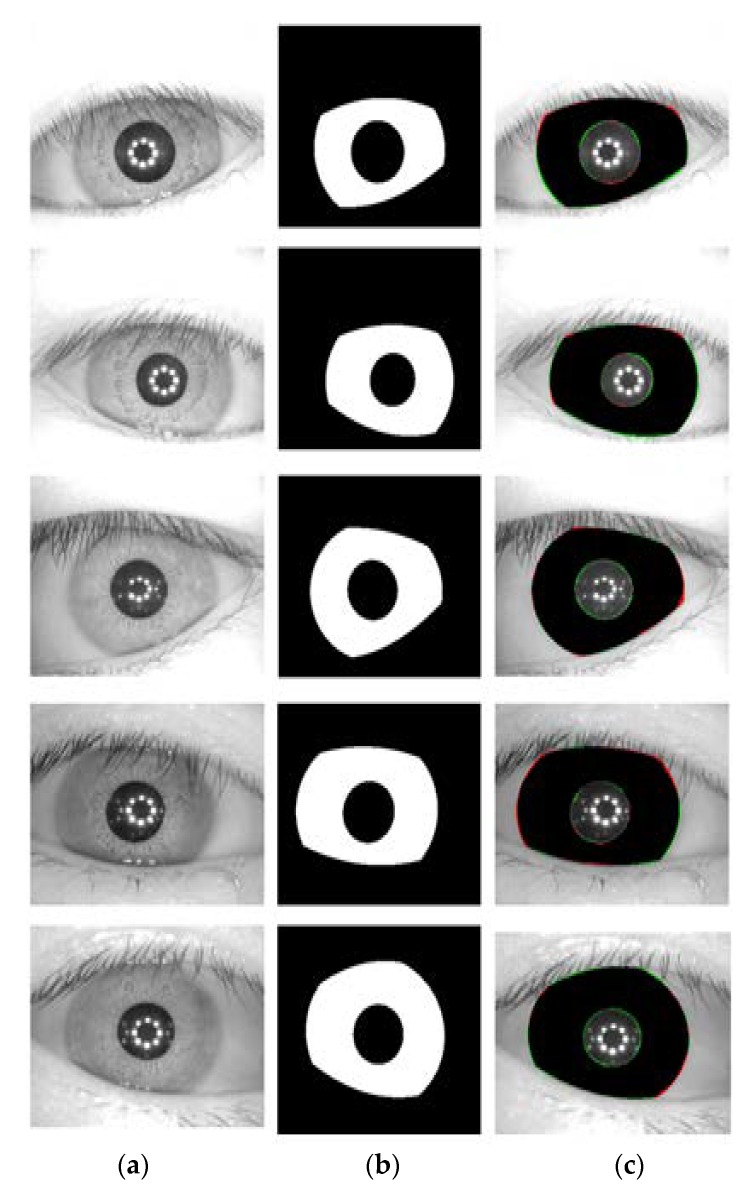
Examples of correct segmentation results in CASIA v4.0 interval database by the proposed method. (**a**) Original image. (**b**) Ground-truth image. (**c**) Segmentation result by IrisDenseNet (The false positive and negative errors are presented as green and red, respectively. The true positive case is presented as black).

**Figure 16:**
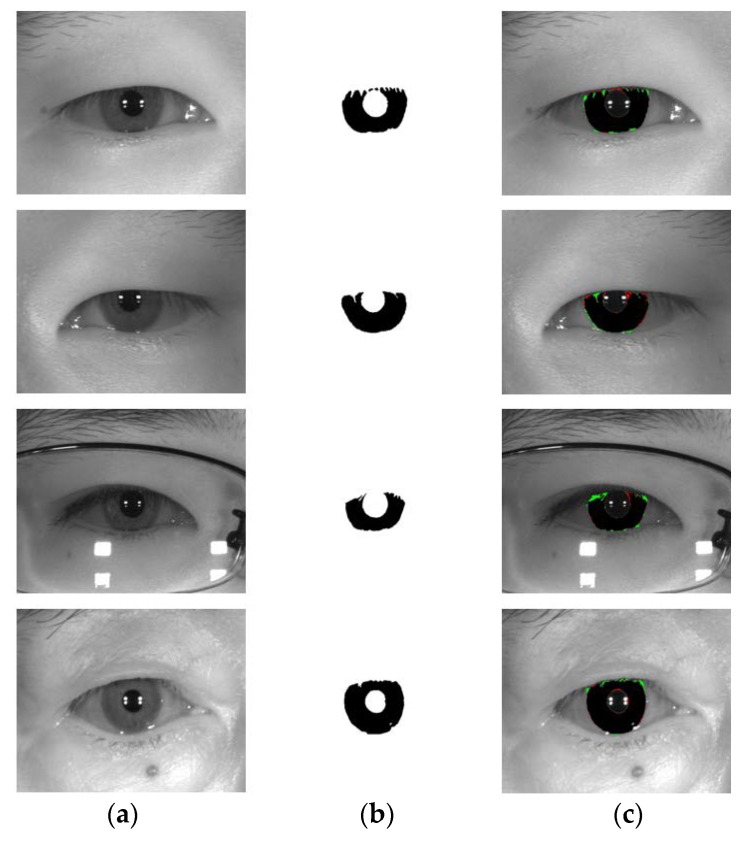
Examples of correct segmentation results in CASIA v4.0 distance database by the proposed method. (**a**) Original image (**b**) Ground-truth image. (**c**) Segmentation result by IrisDenseNet (The false positive and negative errors are presented as green and red, respectively. The true positive case is presented as black).

**Figure 17:**
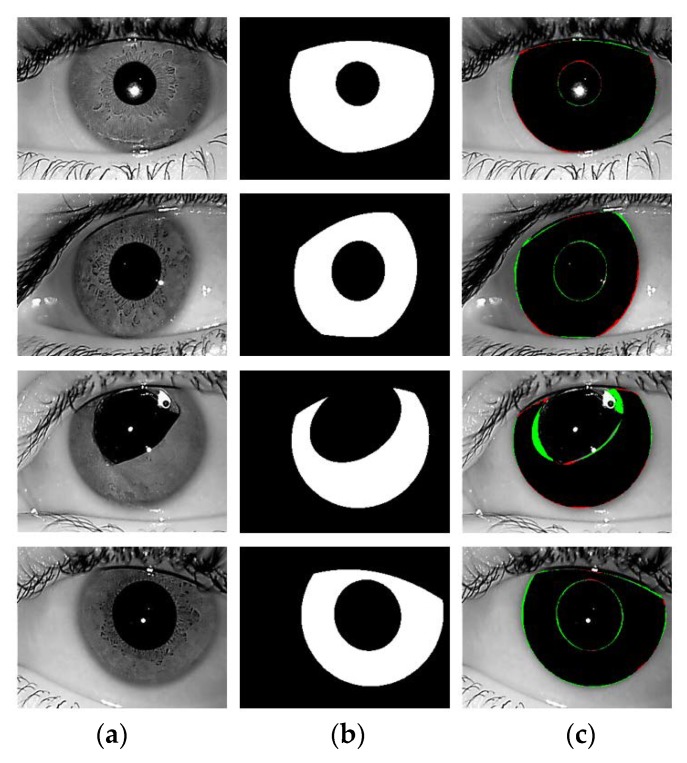
Examples of correct segmentation results in IITD database by the proposed method. (**a**) Original image. (**b**) Ground-truth image. (**c**) Segmentation result by IrisDenseNet (The false positive and negative errors are presented as green and red, respectively. The true positive case is presented as black).

Figure 14, Figure 15, Figure 16 and Figure 17 show the good segmentation results for MICHE-I, CASIA v4.0 interval, CASIA 4.0 distance, and IITD databases, respectively. The false positive and negative errors are presented as green and red, respectively. The true positive is presented as black.

**Figure 18:**
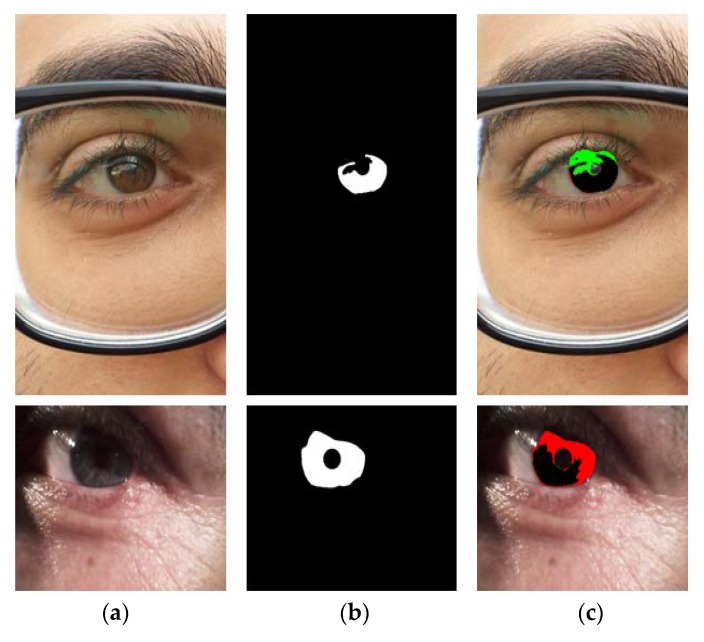
Examples of MICHE-I incorrect segmentation results by our method. (**a**) Original image. (**b**) Ground-truth image. (**c**) Segmentation result by IrisDenseNet (The false positive and negative errors are presented as green and red, respectively. The true positive case is presented as black).

We show the examples of bad segmentation results by IrisDenseNet only for MICHE-I in Figure 18 because there is no considerable error found in other datasets. The false positive errors are caused by reflection noise created by environmental light, whereas the false negative errors are caused by severely dark iris values.

Table 5 and Table 6 show the comparisons of accuracies with MICHE-I and CASIA v4.0 distance databases, respectively, based on the NICE-I evaluation protocol. As shown in Table 5 and Table 6, our method outperforms previous methods. The reason why SegNet-Basic shows lower accuracy than our IrisDenseNet is that the scheme of re-using features through dense connections is not adopted in SegNet-Basic.

To evaluate the accuracies with CASIA v4.0 interval and IITD databases by fair comparative analysis with other researchers for the same datasets, another evaluation protocol is used, which is named as RPF-measure protocol, which was used in [82] for evaluating the iris segmentation performance. The RPF measure is basically recall (R), precision (P), and F-measure, which is similarly used to evaluate the performance of an algorithm based on ground-truth images. The RPF measure is a better way to measure the strength and weakness of an algorithm; to measure from this protocol, the essentials, true positive (*TP*), false positive (*FP*), and false negative (*FN*), should be calculated. Based on them, RPF measure can be computed by Equations (4)–(6), where #*TP*, #*FN*, and #*FP* represent the numbers of *TP*, *FN*, and *FP*, respectively.

(4) $$R=\frac{\#TP}{\#TP+\#FN}$$

(5) $$P=\frac{\#TP}{\#TP+\#FP}$$

(6) $$F=\frac{2\mathrm{RP}}{R+P}$$

where F-measure is basically the harmonic mean of both R and P, and prevents the evaluation from overfitting or underfitting of iris pixels. The comparisons with CASIA v4.0 interval and IITD databases are conducted based on the RPF-measure protocol. However, very few researchers have addressed these two databases for segmentation purposes due to unavailability of ground-truth images. To compare with other researchers, Gangwar et al. [84] used various algorithms such as GST [85], Osiris [86], WAHET [87], IFFP [88], CAHT [89], Masek [90], and integro-differential operator (IDO) [25] with the same database. Therefore, for comparison, the comparative results with these algorithms are presented in Table 7, and it can be found that the proposed IrisDenseNet outperforms other methods. The reason why SegNet-Basic shows lower accuracy than our IrisDenseNet is that the scheme of re-using features through dense connections is not adopted in SegNet-Basic.

## 6. Discussion and Analysis

### 6.1. Power of Dense Connectivity

In this research, the dense connectivity between the layers for better semantic segmentation is used. The conventional methods eliminate the spatial information during the continuous layer by convolution and this continuous elimination of spatial acuity is not good for minor high frequency features in the image. Therefore, the decent way is to import the features from the previous layers to maintain high frequency component during convolution. This maintenance of features from previous layers is done by direct dense connection as shown in Figure 4.

IrisDenseNet is a densely connected fully convolutional network that proceeds in the feed-forward fashion with dense features for better performance. The above discussion about dense features is related to the theoretical discussion, and in this section, a practical comparison of the simple feature with the dense feature is performed using visual images from the convolutional layers. Note that if we compare SegNet with IrisDenseNet in terms of architecture, we can find one unique similarity that both networks use pooling indices for up-sampling, as shown in Figure 2 and Figure 4. Therefore, a careful analysis of the IrisDenseNet network shows that each dense block is separated with a transition layer that has the same pooling layer for pooling indices. Therefore, the difference between the simple and dense features can be fairly compared with each dense block just before the pooling.

**Figure 19:**
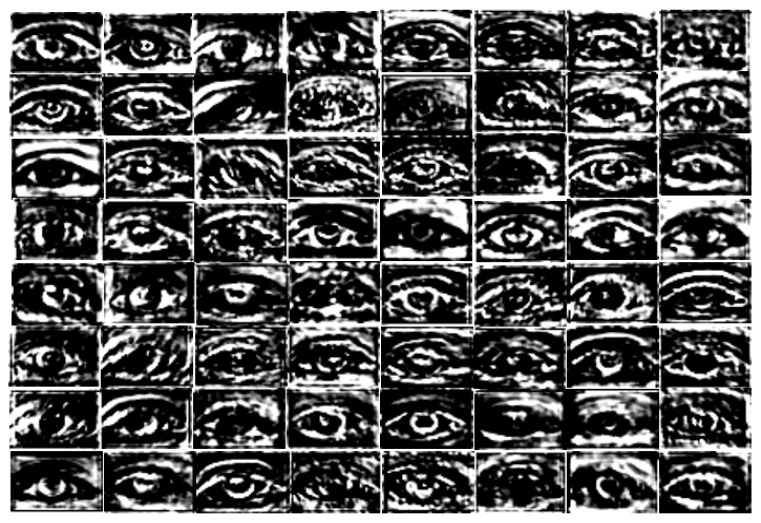
SegNet-Basic last 64 channel (the 449th to 512th channels) features before the 4th max-pooling (Pool-4).

**Figure 20:**
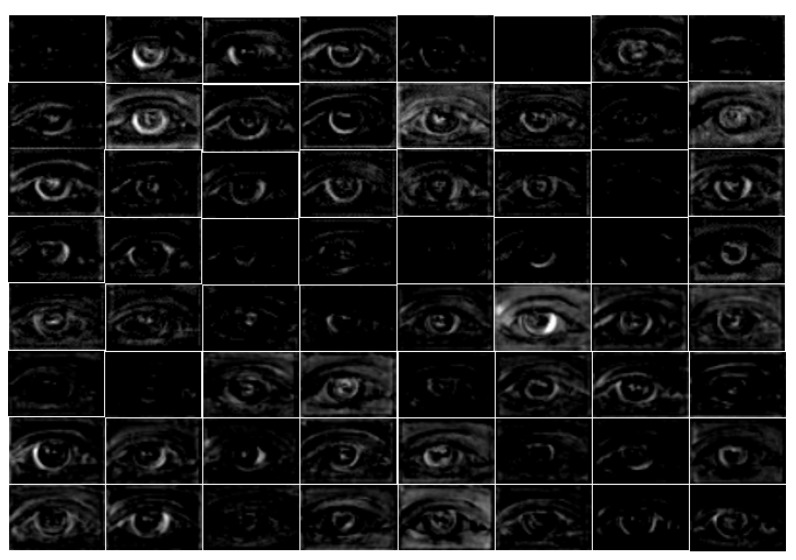
IrisDenseNet last 64 channel (the 449th to 512th channels) features for the 4^th^ dense block before the 4th max-pooling (Pool-4 of Table 2).

In this study, to explain the power of dense connectivity, the reference convolutional features are compared. These are reference features obtained before the 4th max-pooling (Pool-4 of Table 2) layer for both SegNet and IrisDenseNet, as shown in Figure 19 and Figure 20. Note that the output features from Pool-4 contain 512 channels, and for simplicity, the last 64 channels (449th to 512th channels) are visualized. These features present noticeable visual difference. With a careful analysis of the output (shown in Figure 19 and Figure 20), following important observations can be deduced:

- The real power of dense connectivity is evident from the visual features before Pool-4 for both SegNet (Figure 19) and IrisDenseNet (Figure 20). The Pool-4 features are the 2nd-last pooling index features. The Pool-4 features from SegNet include much more noises than those from IrisDenseNet, which can reduce the error of detecting correct iris pixels.

### 6.2. Comparison of Segmentation Results (IrisDenseNet vs. SegNet)

IrisDenseNet uses the concept of feature reuse, which strengthens the features in a dense block for better segmentation. When comparing the SegNet iris segmentation results with IrisDenseNet results, the following differences are found.

**Figure 21:**
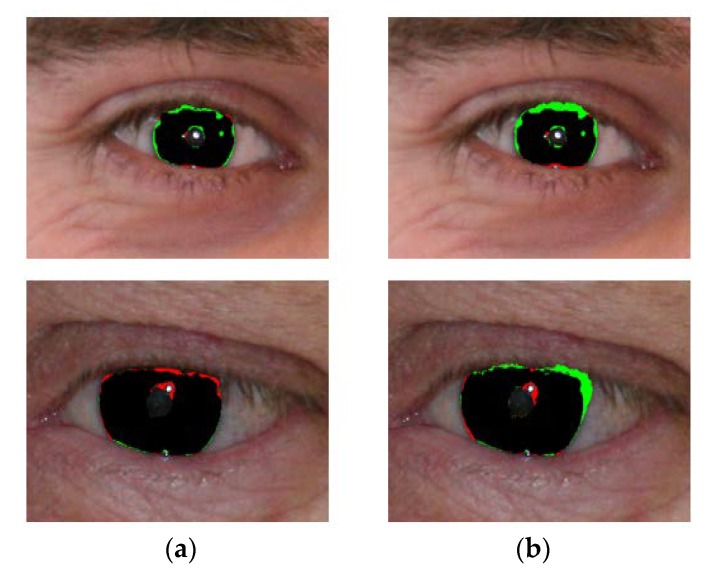
Comparison of iris thin boundary. Segmentation results obtained by (**a**) IrisDenseNet and (**b**) SegNet.

**Figure 22:**
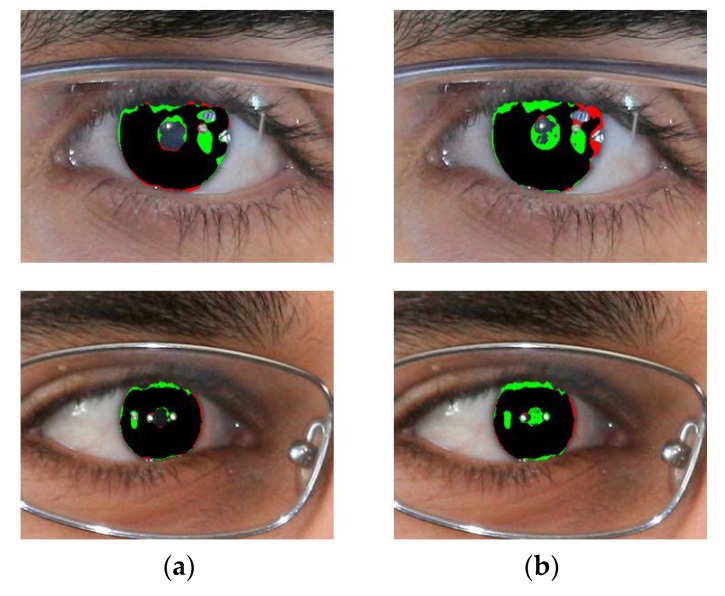
Comparisons of pupil boundary detection. Segmentation results obtained by (**a**) IrisDenseNet and (**b**) SegNet.

**Figure 23:**
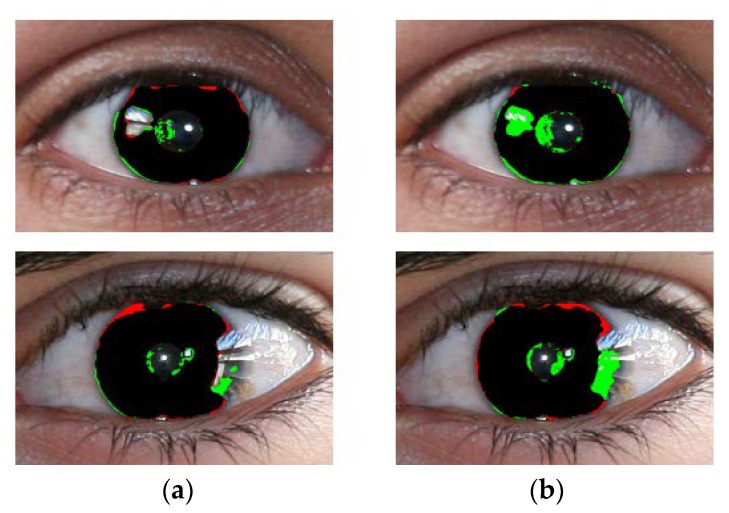
Comparisons of iris detection affected by ghost effect. Segmentation results obtained by (**a**) IrisDenseNet and (**b**) SegNet.

- The segmentation results obtained by IrisDenseNet dense features show a thinner and finer iris boundary as compared to SegNet, which substantially reduces the error rate for the proposed method, as shown in Figure 21a,b.
- IrisDenseNet is more robust for the detection of the pupil boundary as compared to SegNet, as shown in Figure 22a,b.
- IrisDenseNet is more robust for the ghost region in the iris area as compared to SegNet, as shown in Figure 23a,b.

## 7. Conclusions

In this study, we proposed a robust IrisDenseNet with dense concatenated features to segment the true iris boundaries in non-ideal environments even with low-quality images. To achieve better segmentation quality, the encoder was empowered with a dense connection in which layers have direct concatenated connections with all preceding layers in a dense block. This connectivity enhances the capability of the network and enables the feature reuse for better performance. The proposed method provides an end-to-end segmentation without any conventional image processing technique. Experiments conducted with five datasets showed that our method achieved higher accuracies for end-to-end iris segmentation than the state-of-the-art methods for both visible and NIR light environments.

Our method was innovatively powered by dense features but still has a deep network. Therefore, it is difficult to be trained with a GPU with low memory and larger mini-batch size. In the future, we would optimize the network further and reduce the number of layers to make it memory-efficient for mobile and handheld devices with reduced parameters and multiplications. In addition, we will use this method for other applications such as finger vein, human body, or biomedical image segmentations.

## Figures and Tables

**Table 1:** Comparisons between the proposed and existing methods for iris segmentation.

Type	Methods	Strength	Weakness
**Iris circular boundary detection without eyelid and eyelash detection**	Iris localization by circular HT [17,22,24]	These methods show a good estimation of the iris region in ideal cases	These types of methods are not very accurate for non-ideal cases or visible light environments
	Integro-differential operator [18]		
	Iris localization by gradient on iris-sclera boundary points [19]	A new idea to use a gradient to locate iris boundary	The gradient is affected by eyelashes and true iris boundary is not found
	The two-stage method with circular moving window [20]	Pupil based on dark color approximated simply by probability	Calculating gradient in a search way is time-consuming
	Radial suppression-based edge detection and thresholding [21]	Radial suppression makes the case simpler for the iris edges	In non-ideal cases, the edges are not fine to estimate the boundaries
	Adaptive thresholding and first derivative-based iris localization [23]	Simple way to obtain the boundary based on the gray level in ideal cases	One threshold cannot guarantee good results in all cases
**Iris circular boundary detection with eyelid and eyelash detection**	Two-circular edge detector assisted with AdaBoost eye detector [26]	Closed eye, eyelash and eyelid detection is executed to reduce error	The method is affected by pupil/eyelid detection error
	Curve fitting and color clustering [27]	Upper and lower eyelid detections are performed to reduce the error	The empirical threshold is set for eyelid and eyelash detection, and still true boundary is not found
	Graph-cut-based approach for iris segmentation [28]	Eyelashes are removed using Markov random field to reduce error	A separate method for each eyelash, pupil, iris detection is time-consuming
	Rotated ellipse fitting method combined with occlusion detection [29]	Ellipse fitting gives a good approximation for the iris with reduced error	Still, the iris and other boundaries are considered as circular
	Three model fusion-based method assisted with Daugman’s method [30]	Simple integral derivative as a base for iris boundaries is a quite simple way	High-score fitting is sensitive in ideal cases, and can be disturbed by similar RGB pixels in the image
**Active contour-based methods**	Geodesic active contours, Chan–Vese and new pressure force active contours [31,32,33,34]	These methods iteratively approximate the true boundaries in non-ideal situations	In these methods, many iterations are required for accuracy, which takes much processing time
**Region growing and watershed methods**	Region growing with integro-differential constellation [35]	Both iris and non-iris regions are identified along with reflection removal to reduce error	The rough boundary is found first and then a boundary refinement process is performed separately
	Region growing with binary integrated intensity curve-based method [36]	Eyelash and eyelid detection is performed along with iris segmentation	The region growing process starts with the pupil circle, so the case of visible light images where the pupil is not clear can cause errors
	Watershed BIRD with seed selection [37]	Limbus boundary detection is performed to separate sclera, eyelashes, and eyelid pixels from iris	Watershed transform shares the disadvantage of over-segmentation, so circle fitting is used further
**Deep-learning-based methods**	HCNNs and MFCNs [44]	This approach shows the lower error than existing methods for non-ideal cases	The similar parts to iris regions can be incorrectly detected as iris points
	Two-stage iris segmentation method using deep learning and modified HT [45]	Better accuracy due to CNN, which is just applied inside the ROI defined in the first stage	Millions of 21 × 21 images are needed for CNN training and pre-processing required to improve the image
	IrisDenseNet for iris segmentation (Proposed Method)	Accurately find the iris boundaries without pre-processing with better information gradient flow. With robustness for high-frequency areas such as eyelashes and ghost regions	Due to dense connectivity, the mini-batch size should be kept low owing to more time required for training

**Table 2:** IrisDenseNet connectivity and output feature map size of each dense block (Conv, BN, and ReLU represent convolutional layer, batch normalization layer, and rectified linear unit layer, respectively. Cat, B-Conv, and Pool indicate concatenation layer, bottleneck convolution layer, and pooling layer, respectively) (Here, dense blocks 1 and 2 have the same number of convolution layers, and dense blocks 3, 4, and 5 have the same number of convolution layers) (Convolutional layers with “*” mean that these layers include BN and ReLU. Transition layers are a combination of max-pooling and B-Conv)

Block	Name/Size	No. of Filters	Output Feature Map Size (Width × Height × Number of Channel)
**Dense Block-1**	Conv-1_1*/3 × 3 × 3	64	300 × 400 × 64
	Conv-1_2*/3 × 3 × 64	64	
	Cat-1	-	300 × 400 × 128
**Transition layer-1**	B-Conv-1/1 × 1	64	300 × 400 × 64
	Pool-1/2 × 2	-	150 × 200 × 64
**Dense Block-2**	Conv-2_1*/3 × 3 × 64	128	150 × 200 × 128
	Conv-2_2*/3 × 3 × 128	128	
	Cat-2	-	150 × 200 × 256
**Transition layer-2**	B-Conv-2/1 × 1	128	150 × 200 × 128
	Pool-2/2 × 2	-	75 × 100 × 128
**Dense Block-3**	Conv-3_1*/3 × 3 × 128	256	75 × 100 × 256
	Conv-3_2*/3 × 3 × 256	256	
	Cat-3	-	75 × 100 × 512
	Conv-3_3*/3 × 3 × 256	256	75 × 100 × 256
	Cat-4	-	75 × 100 × 768
**Transition layer-3**	B-Conv-3/1 × 1	256	75 × 100 × 256
	Pool-3/2 × 2	-	37 ×50 × 256
**Dense Block-4**	Conv-4_1*/3 × 3 × 256	512	37 ×50 × 512
	Conv-4_2*/3 × 3 × 512	512	
	Cat-5	-	37 ×50 × 1024
	Conv-4_3*/3 × 3 × 512	512	37 ×50 × 512
	Cat-6	-	37 ×50 × 1536
**Transition layer-4**	B-Conv-4/1 × 1	512	37 ×50 × 512
	Pool-4/2 × 2	-	18 × 25 × 512
**Dense Block-5**	Conv-5_1*/3 × 3 × 512	512	18 × 25 × 512
	Conv-5_2*/3 × 3 × 512	512	
	Cat-7	-	18 × 25 × 1024
	Conv-5_3*/3 × 3 × 512	512	18 × 25 × 512
	Cat-8	-	18 × 25 × 1536
**Transition layer-5**	B-Conv-5/1 × 1	512	18 × 25 × 512
	Pool-5/2 × 2	-	9 × 12 × 512

**Table 3:** Comparative accuracies according to various augmentation methods.

Method	$E_{a}$
Excessive data augmentation	0.00729
Data augmentation by changing the contrast and brightness of iris image	0.00761
Proposed data augmentation in Section 5.2	0.00695

**Table 4:** Comparisons of the proposed method with previous methods using NICE-II dataset.

Method	$E_{a}$
Luengo-Oroz et al. [69]	0.0305
Labati et al. [70]	0.0301
Chen et al. [71]	0.029
Jeong et al. [26]	0.028
Li et al. [72]	0.022
Tan et al. [73]	0.019
Proença et al. [74]	0.0187
de Almeida [75]	0.0180
Tan et al. [76]	0.0172
Sankowski et al. [77]	0.016
Tan et al. [35]	0.0131
Haindl et al. [78]	0.0124
Zhao et al. [79]	0.0121
Arsalan et al. [45]	0.0082
SegNet-Basic [57]	0.00784
Proposed IrisDenseNet	0.00695

**Table 5:** Comparison of the proposed method with previous methods using MICHE-I dataset based on NICE-I evaluation protocol.

Method		$E_{a}$
Hu et al. [30]	Sub-dataset by iPhone5	0.0193
	Sub-dataset by Galaxy S4	0.0192
Arsalan et al. [45]	Sub-dataset by iPhone5	0.00368
	Sub-dataset by Galaxy S4	0.00297
	Sub-dataset by Galaxy Tab2	0.00352
SegNet-Basic [57]	Sub-dataset by iPhone5	0.0025
	Sub-dataset by Galaxy S4	0.0027
	Sub-dataset by Galaxy Tab2	0.0029
Proposed IrisDenseNet	Sub-dataset by iPhone5	0.0020
	Sub-dataset by Galaxy S4	0.0022
	Sub-dataset by Galaxy Tab2	0.0021

**Table 6:** Comparison of the proposed method with previous methods using CASIA v4.0 distance database based on NICE-I evaluation protocol.

Method	$E_{a}$
Tan et al. [73]	0.0113
Liu et al. [44] (HCNNs)	0.0108
Tan et al. [76]	0.0081
Zhao et al. [79]	0.0068
Liu et al. [44] (MFCNs)	0.0059
SegNet-Basic [57]	0.0044
Proposed IrisDenseNet	0.0034

**Table 7:** Comparison of the proposed method with previous methods using CASIA v4.0 interval and IITD databases based on the RPF-measure evaluation protocol. A smaller value of *σ* and a higher value of *µ* show better performance. (unit: %) (The resultant values of GST [85], Osiris [86], WAHET [87], IFFP [88], CAHT [89], Masek [90], IDO [25], and IrisSeg [84] are referred from [84]).

DB	Method	R		P		F	
		μ	σ	μ	σ	μ	σ
**CASIA V4.0 Interval**	GST [85]	85.19	18	89.91	7.37	86.16	11.53
	Osiris [86]	97.32	7.93	93.03	4.95	89.85	5.47
	WAHET [87]	94.72	9.01	85.44	9.67	89.13	8.39
	IFFP [88]	91.74	14.74	83.5	14.26	86.86	13.27
	CAHT [89]	97.68	4.56	82.89	9.95	89.27	6.67
	Masek [90]	88.46	11.52	89	6.31	88.3	7.99
	IDO [25]	71.34	22.86	61.62	18.71	65.61	19.96
	IrisSeg [84]	94.26	4.18	92.15	3.34	93.1	2.65
	SegNet-Basic [57]	99.60	0.66	91.86	2.65	95.55	1.40
	Proposed Method	97.10	2.12	98.10	1.07	97.58	0.99
**IITD**	GST [85]	90.06	16.65	85.86	10.46	86.6	11.87
	Osiris [86]	94.06	6.43	91.01	7.61	92.23	5.8
	WAHET [87]	97.43	8.12	79.42	12.41	87.02	9.72
	IFFP [88]	93.92	10.62	79.76	11.42	85.83	9.54
	CAHT [89]	96.8	11.2	78.87	13.25	86.28	11.39
	Masek [90]	82.23	18.74	90.45	11 .85	85.3	15.39
	IDO [25]	51.91	15.32	52.23	14.85	51.17	13.26
	IrisSeg [84]	95.33	4.58	93.70	5.33	94.37	3.88
	SegNet-Basic [57]	99.68	0.51	92.53	2.05	95.96	1.04
	Proposed Method	98.0	1.56	97.16	1.40	97.56	0.84
